# Accurate Identification Partial Discharge of Cable Termination for High-Speed Trains Based on S-Transform and Two-Dimensional Convolutional Network Algorithm

**DOI:** 10.3390/s24237602

**Published:** 2024-11-28

**Authors:** Yunlong Xie, Peng You, Guangning Wu, Tingyu Zhang, Yang Luo, Shuyuan Zhou, Kai Liu, Kui Chen, Dongli Xin, Guoqiang Gao

**Affiliations:** 1 School of Electrical Engineering, Southwest Jiaotong University, Chengdu 611756, China; yunlongxie@my.swjtu.edu.cn (Y.X.); pengy@my.swjtu.edu.cn (P.Y.); gnwu@swjtu.edu.cn (G.W.); tingyu@my.swjtu.edu.cn (T.Z.); lluo@my.swjtu.edu.cn (Y.L.); zsyuan@my.swjtu.edu.cn (S.Z.); xindonglihero@my.swjtu.edu.cn (D.X.); xnjdggq@swjtu.edu.cn (G.G.); 2 Tangshan Institute, Southwest Jiaotong University, Tangshan 063000, China

**Keywords:** cable termination, partial discharge, signal identification, convolutional neural networks, wavelet transform

## Abstract

Cable termination is an important part of energy transmission in high-speed trains, and it is also a weak link in the insulation. It is important to determine the insulation status of cable terminals by the detection results of partial discharge signals, but the partial discharge signals in the field test circuit are mixed with a large amount of external corona interference, which affects the detection accuracy. This paper proposes a signal recognition model that incorporates Stockwell transform (ST) and 2DCNN, which in combination with wavelet noise reduction can achieve a high-precision classification effect for partial discharge and corona interference with an accuracy rate of up to 98.75%. By selecting the maximum energy moment in the ST matrix to correct the position of the time window during the recognition of long time series signals, the problem of corona interference being truncated by the time window and being misidentified as partial discharge is overcome, and the generalization ability of the model is enhanced. Experimental results show that the method has an excellent performance in separating partial discharge and corona interference in long time series signals.

## 1. Introduction

With the rapid development of the national economy and the continuous improvement of national defense and military security needs, “high speed, large volume, low cost, long distance, high reliability” has become the development direction of high-speed rail transportation and ensuring the safe and stable service performance of high-speed EMUs has become the core concern of the rail transit industry. As the key carrier of energy supply, transmission and conversion of high-speed trains, vehicle-mounted cable is the only channel for EMU power transmission, which plays a decisive role in ensuring the safety and stability of train operation [1,2,3,4]. The insulation capacity of the vehicle cable terminal is relatively weak, and it is prone to permeable interlayer surface discharge due to long-term interference by factors such as high temperature, high pressure, pollution erosion and mechanical vibration in the external environment, resulting in insulation breakdown [5,6,7,8]. The interfacial air gap is a defect that often occurs inside the cable termination, and its generation and presence can lead to partial discharge (PD) [9,10]. Partial discharge is an important method to detect the insulation state of cable terminals, and the degree of deterioration of insulation can be judged according to the detected partial discharge, and then maintenance or replacement measures can be taken in time.

Partial discharge detection is a key means to evaluate the insulation state of the cable terminal. By monitoring partial discharge, the degree of insulation and deterioration can be assessed [11,12,13], and the cable terminations can be maintained or replaced in a timely manner [14,15,16]. Usually, when partial discharge occurs inside the cable terminal, it will be accompanied by the generation of ultrasonic, impulse voltage, impulse current, heat, light and electromagnetic waves. According to the various phenomena produced by partial discharge, many corresponding detection methods have been researched, which can be classified into electrical and non-electrical quantity detection methods [17,18]. At present, electrical quantity detection methods mainly include the pulse current method, high-frequency current method (HFCT), and ultra-high-frequency method. Non-electrical detection methods mainly include the ultrasonic detection method, light detection method, temperature detection method, etc. [19,20]. The HFCT has the advantages of easy installation, simple structure, no impact on the operation status of cable terminals, high test sensitivity, strong anti-interference ability, and quantitative analysis of partial discharge signals, etc., but the disadvantage is that it is susceptible to the influence of the grounding wire cycle, and it is mainly used for the partial discharge measurement of high-voltage cables and electrical equipment [21,22,23].

During on-site measurements, tip parts such as pantographs will cause corona discharge (CD). As a pulse-type interference, CD will affect the fault judgment of high-speed trains [24,25,26]. Separating the pulse interference in the measurement signal avoids misjudgments in fault detection and the identification of partial discharge sources and allows for a more reliable diagnosis of the fault mechanisms involved in vehicle-mounted cable terminations [27,28,29,30]. Based on the statistical operator and vector norm of phase-resolved partial discharge (PRPD), Masoud Karimi extracted the features of the measured raw discharge signal, studied the influence of the window interval on the classification accuracy in the PRPD method, found the window size with better classification accuracy, and used the deep belief neural network to classify corona discharge, surface discharge and internal discharge [31,32]. Chang used wavelet packet transform to extract the energy tree, kurtosis tree and skewness tree as features for partial discharge, corona discharge and mixed signal, and they used a three-layer feedforward neural network with backpropagation learning rule as a classifier to effectively remove corona discharge [33]. Raymond extracted the PRPD image of the PD signal as the input of the convolutional neural network to realize the classification of the PD signal [34]. Neural network algorithms have made effective progress in the partial discharge pattern recognition of power equipment, among which the convolutional neural network (CNN) efficiently extracts features through convolutional layers and pooling layers, which is suitable for local feature extraction and pattern recognition and performs well in image classification [35,36,37,38,39,40].

Aiming at the problem of partial discharge signal and corona interference identification, some scholars have established a one-dimensional convolutional neural network model, used the trained CNN for the suppression of corona interference in the long-time series signal after noise reduction, added a time window to scan the long-time series signal, and intercepted the signal and identified it at the same time [41,42]. The results show that the proposed method can preliminarily identify the PD signal from the mixed signal, and the measured signal at different voltage levels is universal. However, there are still shortcomings in this method. In practical applications, corona interference will be truncated by the time window, resulting in the corona interference signal being incorrectly identified as a partial discharge signal.

In this paper, the partial discharge recognition model of vehicle-mounted cable terminals is improved, and a convolutional neural network (WT-ST-2DCNN) signal classification model based on wavelet transform and Stockwell transform (ST) is proposed, which removes the noise in the signal through wavelet noise reduction, introduces ST to analyze the time frequency of the signal, and constructs a two-dimensional convolutional neural network model, which can separate the partial discharge and corona interference in the long-time sequence signal of the vehicle-mounted cable terminal, realizing the accurate classification and identification of PD and CD.

## 2. Data Acquisition

### 2.1. Experimental Platform Construction

**Figure 1:**
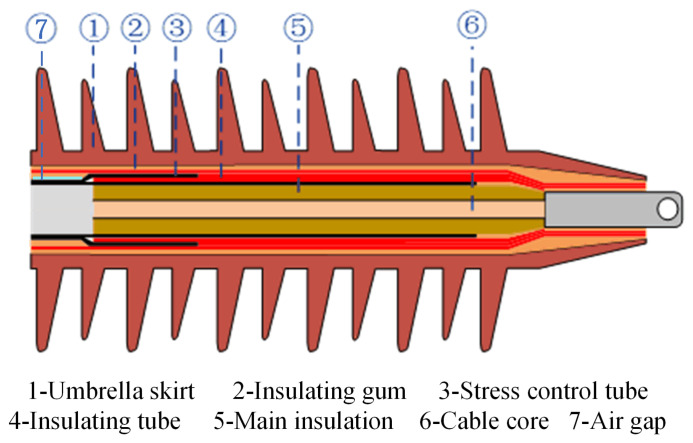
Structure of vehicle-mounted cable terminal [34].

The high-frequency partial discharge test is carried out in the high-voltage test hall, and to simulate the discharge fault when there is an air gap between the main insulation and stress control tube, it is necessary to fabricate cable terminations with air gap defects. The air gap is 50 mm long and 2 mm wide, which traps the steel needle inside the cable termination, ensuring the integrity of the air gap defect. The cable termination structure is shown in Figure 1.

The CD phenomenon is caused by excessive local field strength due to distortion of the electric field, which usually occurs at the sharp edge of the surface of the cable termination and usually does not cause failure. Therefore, for the acquisition of CD signals, the anti-halo ball can be pinned to increase the field strength distortion of the high-voltage terminal and corona discharge. For PD signals, only an anti-corona ball is installed at the high-voltage terminal to prevent CD from occurring.

**Figure 2:**
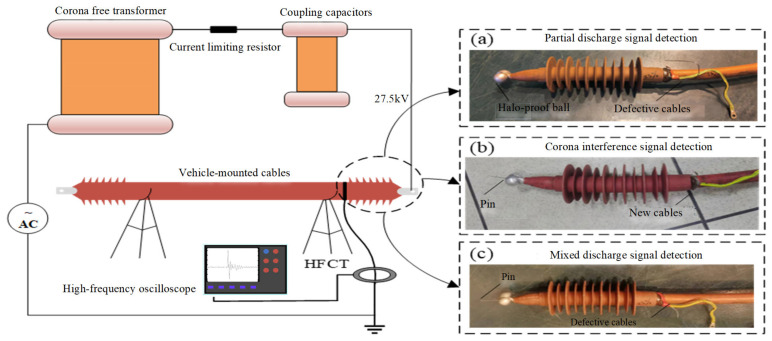
Signal acquisition scheme based on HFCT.

**Figure 3:**
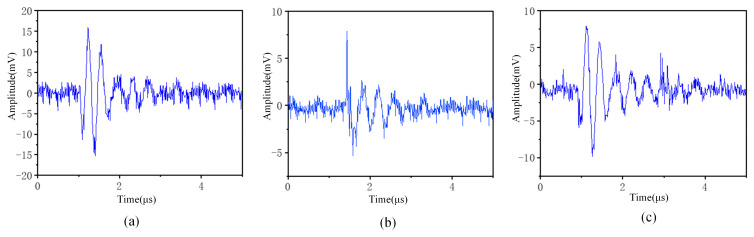
Typical signal. (**a**) Partial discharge. (**b**) Corona interference. (**c**) Mixed signal [34].

The laboratory setup for HFCT test loop is shown in Figure 2 with a test voltage level of 27.5 kV. In the test loop above, an anti-halo ball is installed at the end of the defective cable to ensure that the measured pure PD signal is generated. The CD signal is measured by pin processing on a halo-proof ball at the high-voltage terminal of a non-defective cable termination. A pin is inserted into the anti-halo ball with defective cable terminations to detect and record the mixed signal of PD and CD. Figure 3 shows the waveforms of PD, CD, and mixed discharges obtained through three experiments.

### 2.2. Signal Denoising

In the laboratory environment, there is white noise interference that affects the original form of the signal. In this paper, the method of wavelet transform is used to remove the white noise in the experimental signal. The basic idea of the wavelet denoising method is to remove the wavelet coefficients corresponding to noise in each frequency band according to the characteristics of different intensity distribution of the wavelet decomposition coefficients of the noise and signal in different frequency bands, retain the wavelet decomposition coefficients of the original signal, and then carry out wavelet reconstruction of the processed coefficients to obtain a pure signal.

**Figure 4:**
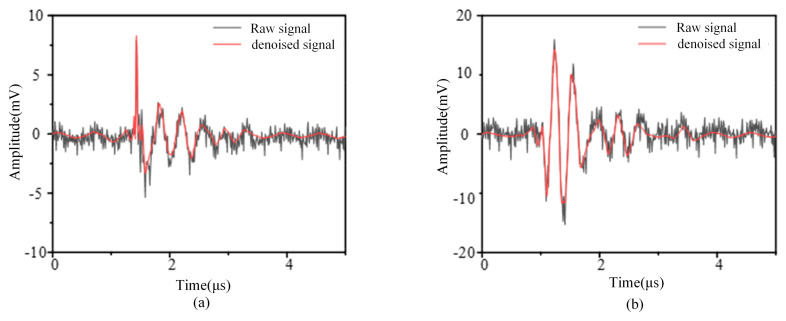
Wavelet denoising result. (**a**) Corona interference signal. (**b**) Partial discharge.

Firstly, the collected experimental signals are decomposed by the wavelet. Since the noise signals are mostly contained in the details with higher frequencies, the wavelet coefficients smaller than the threshold are zeroed out by setting different thresholds for the high-frequency components of each layer, and then the PD and CD after noise removal can be obtained by using the threshold wavelet coefficients for wavelet reconstruction. Drawing from reference [12], sym4 is selected as the wavelet basis function in this paper, and the number of decomposition layers is 8. The white noise in PD and CD signals can be effectively filtered and burrs can be removed by wavelet transform. The experimental signals after noise removal are shown in Figure 4a,b.

## 3. Partial Discharge Recognition of Vehicle Cable Terminal Based on WT-ST-2DCNN

In order to solve the problems of the traditional neural network model in long-time signal recognition, three time–frequency analysis methods are compared in this paper, and the combination of ST and 2DCNN is selected. The recognition effect of ST-2DCNN before and after wavelet noise reduction is compared, and the method proposed in this paper is compared with other methods, which proves the superiority of the signal recognition model proposed in this paper. In the identification of mixed signals in long-time series signals, the time–frequency performance of ST is used to correct the position of the time window, which improves the application performance of 2DCNN in separating partial discharge signals from corona interference in long-time signals at vehicle cable terminals.

### 3.1. Time–Frequency Analysis Methods

ST combines the time–frequency analysis idea of short-time Fourier transform (STFT) and the ability of continuous wavelet transform (CWT) to process signals, allowing the window width to change with time, providing different frequency resolutions in different time periods. In addition, it can also distinguish high-frequency and low-frequency information in the information at the same time, which can better adapt to the non-stationary characteristics of the signal. For any one-dimensional time-series signal *h*(*t*), the S transformation formula is as follows:

(1) $$\mathrm{ST}(\tau ,f)=\int_{-\infty }^{+\infty }h(t)w(\tau -t,f)e^{-i2\pi ft}dt$$

where *w*(*τ* − *t*, *f*) is the Gaussian window, and *τ* is the parameter that controls the position of the Gaussian window on the t-axis. It can be seen from the equation that the S transform is different from the short-time Fourier transform in that the height and width of the Gaussian window change with frequency, thus overcoming the defect of fixed height and width of the short-time Fourier transform window.

### 3.2. Establishment of Partial Discharge Time–Frequency Mapping Dataset for Vehicle-Mounted Cable Terminal

#### 3.2.1. Time–Frequency Analyses Based on Different Methods

**Figure 5:**
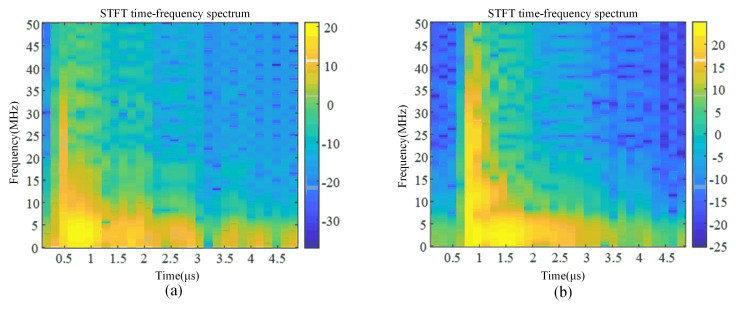
Signal time–frequency spectrum based on STFT. (**a**) Time–frequency spectrum of partial discharge signal. (**b**) Time–frequency spectrum of corona discharge signal.

**Figure 6:**
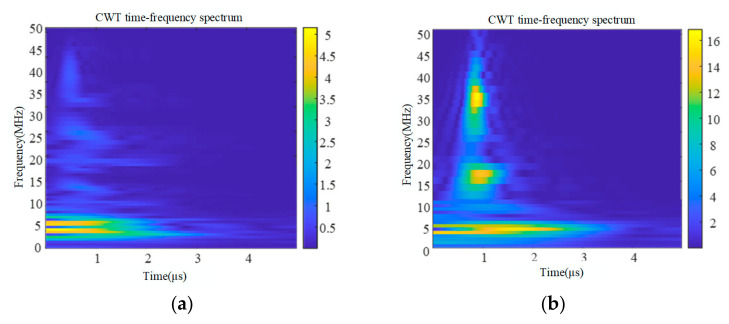
Signal time–frequency spectrum based on CWT. (**a**) Time–frequency spectrum of partial discharge signal. (**b**) Time–frequency spectrum of corona discharge signal.

**Figure 7:**
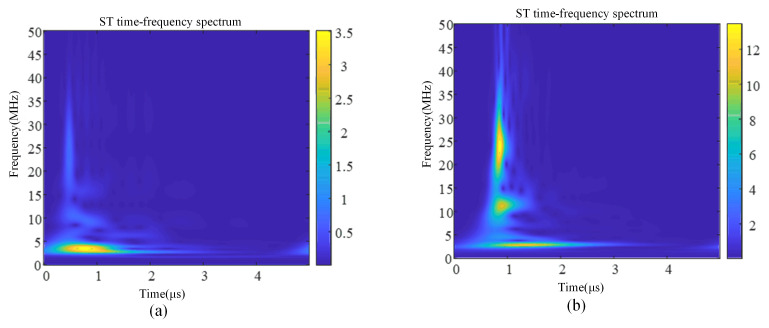
Signal time–frequency spectrum based on ST. (**a**) Time–frequency spectrum of partial discharge signal. (**b**) Time–frequency spectrum of corona discharge signal.

The partial discharge and corona interference signals measured in the laboratory were selected with a signal length of 5 μs, and the STFT time–frequency transformation, CWT time–frequency transformation, and ST time–frequency transformation were carried out for these two signals, respectively, and the results are shown in Figure 5, Figure 6 and Figure 7.

By comparing the time–frequency spectra of the three time–frequency analysis methods, it can be obtained that although STFT and CWT can also obtain time–frequency analysis spectra, their frequency aggregation is low, and the resolution of STFT is single. The window function of the ST varies with frequency: the wide time window is used at low frequency and the narrow time window is used at high frequency, which obtains a higher time–frequency aggregation degree, improves the time–frequency localization accuracy, and has a higher time–frequency resolution compared with STFT and CWT. The ST not only overcomes the problem of fixed time–frequency resolution in the short-time Fourier transform but also effectively avoids the problem of wavelet basis and decomposition layer selection in the wavelet transform, which is very suitable for dealing with non-stationary time series signals. Therefore, the ST is selected as the time–frequency analysis method for partial discharge and corona interference.

#### 3.2.2. Dataset Generation of Time–Frequency Spectrum

**Figure 8:**
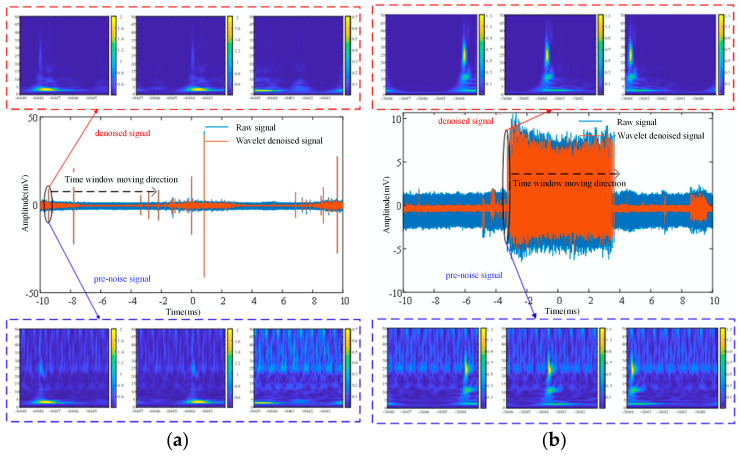
Time-frequency spectrum acquisition. (**a**) Time-frequency spectrum acquisition of partial discharge signal based on ST. (**b**) Time-frequency spectrum acquisition of corona discharge signal based on ST.

The signal measured in the laboratory is usually a signal under a period of [−10, 10] ms. A time window of 5 μs is set at the beginning of the signal, and the sliding step is 2 μs. The results of the partial discharge and corona disturbances scanned separately for the entire period are shown in Figure 8. As can be seen from Figure 8, multiple time–frequency spectra can be obtained in different time windows for signals at the same time, which can provide more diverse inputs for the model and help improve the generalization ability of the model. In a time–frequency spectrum, color indicates the energy intensity of the signal, while frequency and time correspond to the longitudinal and transverse aspects of the spectrum, respectively. Subtle differences in the time–frequency maps obtained at different time windows were observed, which reflected the dynamic changes of the signal at different time scales. By introducing this diversity, the model can better understand the time–frequency characteristics of the signal, allowing for more accurate signal identification.

At the same time, the red dotted box is the time–frequency spectrum after noise reduction, and the blue dotted box is the time–frequency spectrum before noise reduction, which can effectively reduce the noise interference in the signal and improve the clarity of the time–frequency characteristics of the target signal in the figure through the noise reduction operation. White noise is a random, uniformly distributed noise that appears in the time–frequency spectrum as a random distribution in frequency and time, and for signals with different amplitudes, it presents different effects on the time–frequency spectrum. By taking advantage of the local nature of the wavelet transform, wavelet noise reduction technology can remove the noise component while retaining the main characteristics of the signal, improving the quality and reliability of the time–frequency spectrum. This can ensure that the model can better capture the real time–frequency characteristics of the signal and remove the redundant time–frequency information in the map, which lays the foundation for the classification and recognition of the later model.

### 3.3. WT-ST-2DCNN Partial Discharge Identification Model

#### 3.3.1. Theoretical Analysis Based on WT-ST-2DCNN

**Figure 9:**
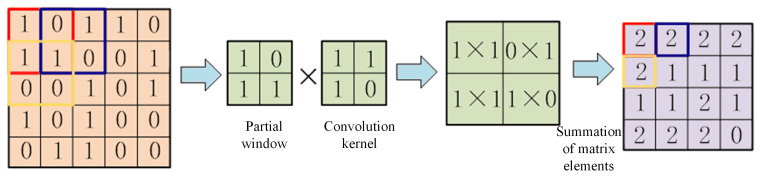
Convolution operation based on 2DCNN.

**Figure 10:**
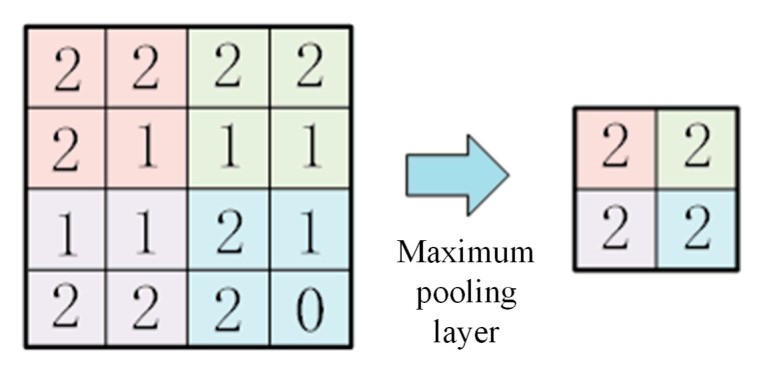
Maximum pooling operation based on 2DCNN.

There are main differences between two-dimensional convolutional neural networks and one-dimensional convolutional neural networks in the processing dimension of data: the convolutional kernel of one-dimensional convolutional neural networks is usually one-dimensional, which can be regarded as a sliding window with a fixed width, moving in the direction of the input sequence, and calculating the inner product to generate the output sequence. The convolutional kernel of a two-dimensional convolution is two-dimensional, usually a matrix, which slides in the row and column directions of the input image and calculates the weighted sum of the local regions to generate the output feature map, as shown in Figure 9. In a one-dimensional convolution, the maximum pooling layer uses a one-dimensional pooling region, and the maximum pooling operation looks for the maximum value in each one-dimensional region of the input sequence and uses it as the corresponding position of the output sequence. In 2D convolution, the maximum pooling layer usually uses a 2D pooling region, and the maximum pooling operation looks for the maximum value in each 2D region of the input feature map and uses it as the corresponding position of the output feature map, as shown in Figure 10.

The combination of the ST and 2DCNN is mainly to solve the above problem that the corona interference in the long-time mixed-signal is truncated by the time window and is misidentified as partial discharge, and the key to solving this problem lies in the ST matrix generated by the ST. From the mathematical expression of the ST, it can be concluded that the ST can decompose the time series signal into a complex time–frequency matrix, that is, the ST matrix, with the matrix rows corresponding to the time axis and the columns corresponding to the frequency axis. The modulus value of each element in the ST matrix is obtained to obtain the time–frequency amplitude matrix of the signal, and the amplitude matrix can visually display the energy distribution of each frequency component of the signal on the time axis. By finding the time point corresponding to the maximum energy, the time window on the long time series signal can be re-corrected.

**Figure 11:**
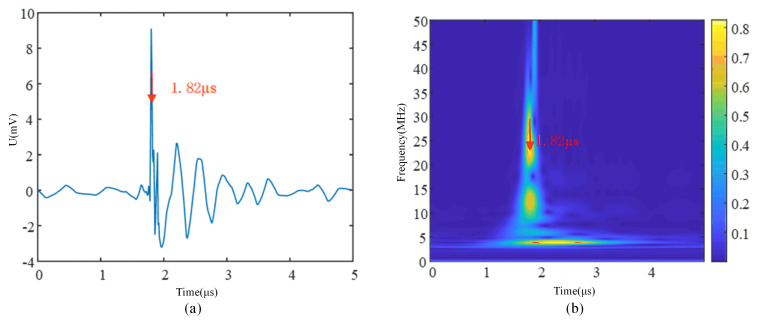
Corona interference spectrum. (**a**) Corona interference waveform diagram. (**b**) Time–frequency spectrum of corona interference.

Taking a single corona interference as an example, as shown in Figure 11b, the time corresponding to the maximum energy in the corona interference is 1.82 μs, which corresponds to the location of 1.82 μs in the corona interference waveform in Figure 11a. With this mapping, the position of the window can be corrected as the time window slides over a long time series signal, ensuring that the complete corona interference is intercepted.

#### 3.3.2. Signal Recognition Process Based on WT-ST-2DCNN

**Figure 12:**
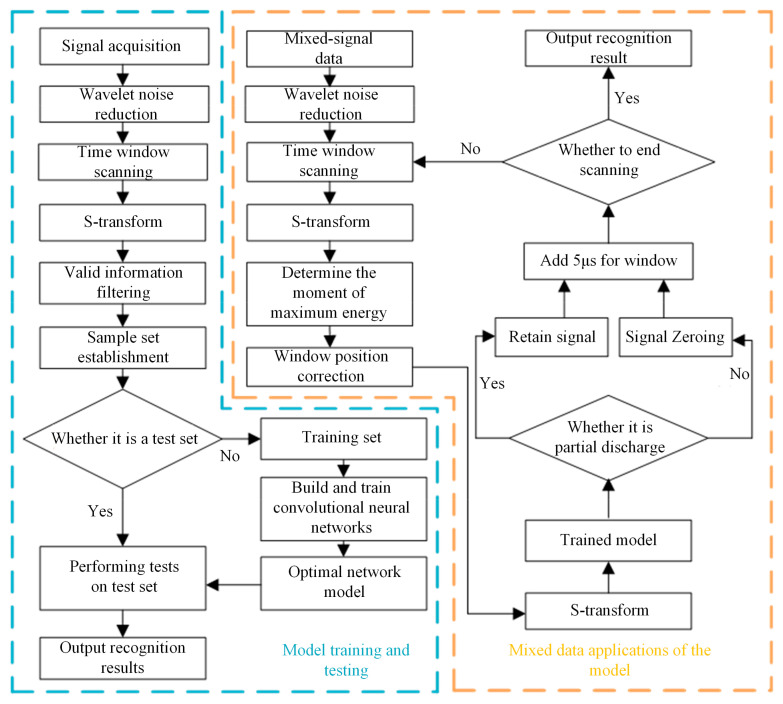
Signal recognition process of ST-2DCNN.

The signal recognition based on WT-ST-2DCNN is divided into two parts: model training and testing and model mixed data application, as shown in Figure 12.

#### 3.3.3. Network Structure and Parameters Based on WT-ST-2DCNN

**Figure 13:**
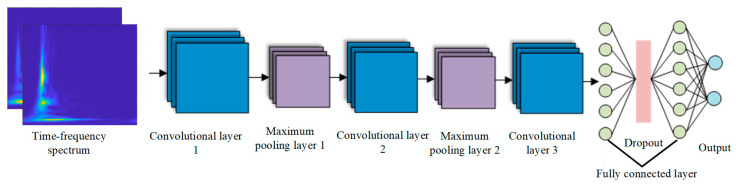
Network structure diagram of 2DCNN.

The 2DCNN model proposed in this section consists of three convolutional layers, three activation layers, two maximum pooling layers, two fully connected layers and one layer of dropout. The network structure is shown in Figure 13, and the specific parameter settings are shown in Table 1. The function of the convolutional layer is to extract the local features of the input data and extract the key feature information through the sliding calculation of the convolutional kernel. The activation layer uses the ReLU function to introduce nonlinear transformation capabilities, enhancing the model’s ability to fit complex data. The maximum pooling layer is used to reduce dimensionality and feature redundancy and at the same time improve the robustness of the model to spatial transformation. The fully connected layer completes the classification task by mapping the extracted features into the classification space. The dropout layer is used to randomly discard some neurons during training, effectively preventing the model from overfitting.

We extracted features from the stacked convolutional layer and the maximum pooling layer, and then we compressed the information into the form of feature mapping. We set the padding amount of each convolutional layer to SAME to ensure that the output size of the convolution operation is the same as the input size and thus avoid the loss of information at the edge of the image during the convolution operation. To improve the training effect of the model, ReLU was selected for the activation function, which can effectively alleviate the gradient vanishing problem and improve the convergence speed of the model. Adding two fully connected layers to the network increases the depth and complexity of the model, which reduces the risk of overfitting the model and allows for more complex representations of features to be learned and better distinguishing between different classes. By introducing regularization techniques such as dropout layers, overfitting can be further reduced, and the generalization ability of the model can be improved. In addition, the Adam optimizer was used in conjunction with the initial value of the learning rate of 0.01, combined with the L2 regularization term of 0.01 and the strategy of reducing the learning rate every 450 iterations, to further optimize the training process of the model and improve the final classification accuracy. The combination of these configuration parameters and optimization techniques helps to train a robust model to effectively accomplish our image classification tasks.

All of the methods used in this article were implemented on MATLAB 2022, which is installed on a personal computer equipped with an Intel i9-12900H CPU with a 2.5 GHz clock rate and 16 GB RAM.

### 3.4. Research on PD Identification Based on WT-ST-2DCNN

#### 3.4.1. Analysis of Identification Results of WT-ST-2DCNN

**Figure 14:**
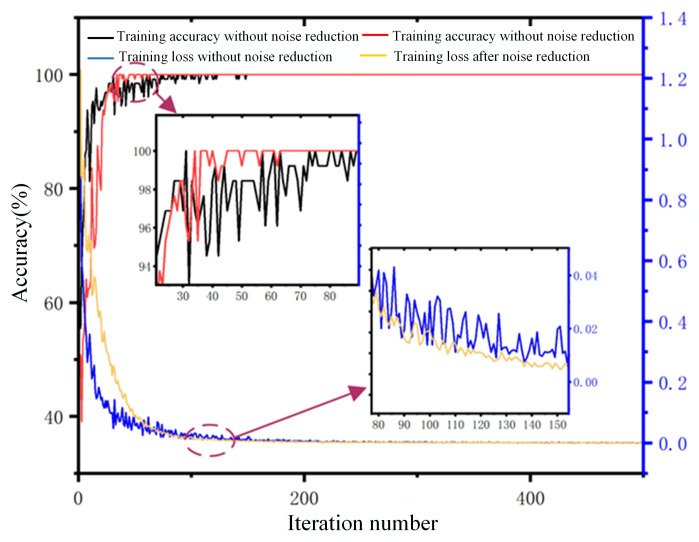
Training loss and accuracy based on data before and after noise reduction.

**Figure 15:**
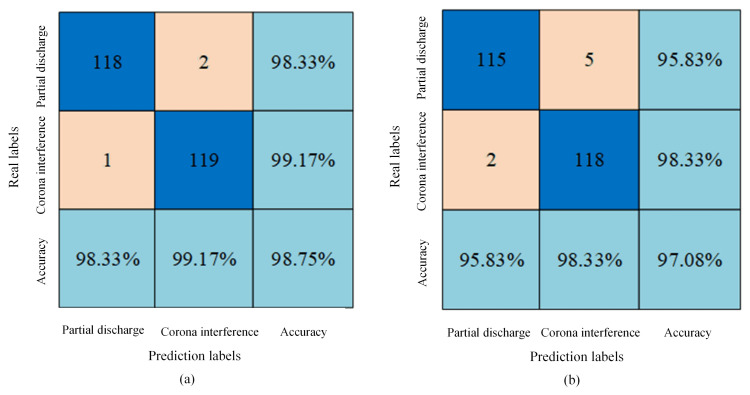
Confusion matrix based on data before and after noise reduction. (**a**) Confusion matrix based on denoised data. (**b**) Confusion matrix based on pre-noise reduction data.

The signal time–frequency spectrum before and after noise reduction was used to make a dataset. The partial discharge and corona interference in each dataset accounted for 600 each, the total data volume was 1200, and the dataset was divided into a training set, a verification set and a test set according to the ratio of 5:3:2. The 2DCNN method was used for classification and identification. The loss and accuracy curves before and after noise reduction were obtained as shown in Figure 14, and the confusion matrix is shown in Figure 15.

As shown in Figure 14, it is found that the loss curve and accuracy curve obtained based on the denoising dataset converge first before the denoising. The test accuracy of the denoised dataset is 97.08%, and the test accuracy of the denoised data is 98.75%. Due to the different amplitudes of the signal, the degree of influence of noise on the time–frequency spectrum is also different, with high amplitude being less affected and low amplitude being greatly affected. Using WT to denoise the signal can better retain the local features of the signal, remove useless information, avoid blurring or confusing information, improve the convergence speed and generalization ability of the model, and achieve higher accuracy on the test set.

#### 3.4.2. Comparison with Other Methods

**Figure 16:**
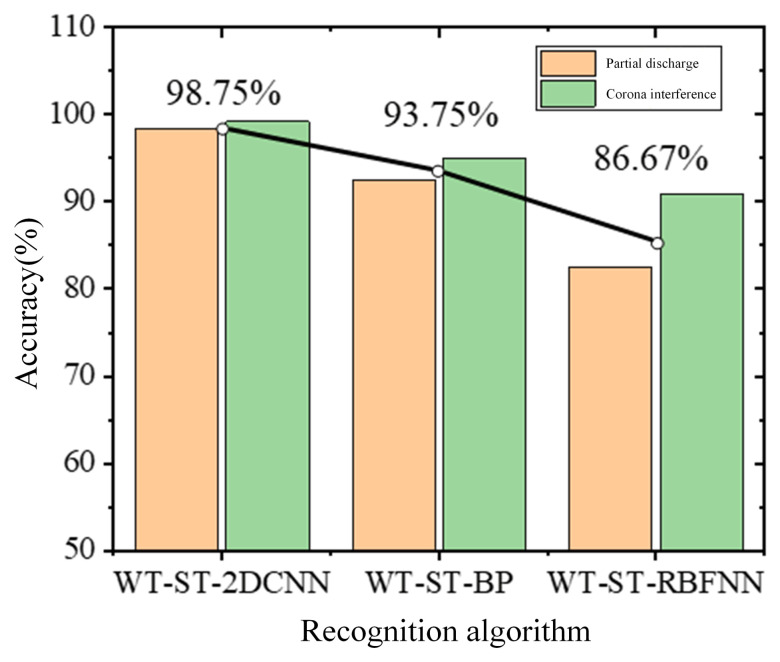
Comparison of different model recognition methods.

In order to prove the superiority of the proposed method, the recognition accuracy of WT-ST-2DCNN is compared with WT-ST-BP and WT-ST-RBFNN, and the recognition accuracy of the three methods is shown in Figure 16.

From the above results, it can be found that the recognition effect of WT-ST-2DCNN is better than that of WT-ST-BP and WT-ST-RBFNN, and the recognition accuracy of partial discharge and corona interference is higher, which can reach more than 98%. RBFNN usually adopts a fully connected structure when processing images, and it uses the radial basis function as the activation function, which has certain nonlinear modeling capabilities, but its training process is relatively simple, and it is inferior to BP and 2DCNN in image processing. The BP neural network is a fully connected feedforward neural network, which is trained by a backpropagation algorithm, and the recognition accuracy also reaches 93.75% in the signal recognition process. However, the 2DCNN proposed in this paper has more advantages in processing image data, because the convolutional layer and pooling layer of 2DCNN can effectively capture the local features in the image, and it has a good ability to extract the hierarchical features of the data.

#### 3.4.3. Separation of Mixed Signals Based on WT-ST-2DCNN

**Figure 17:**
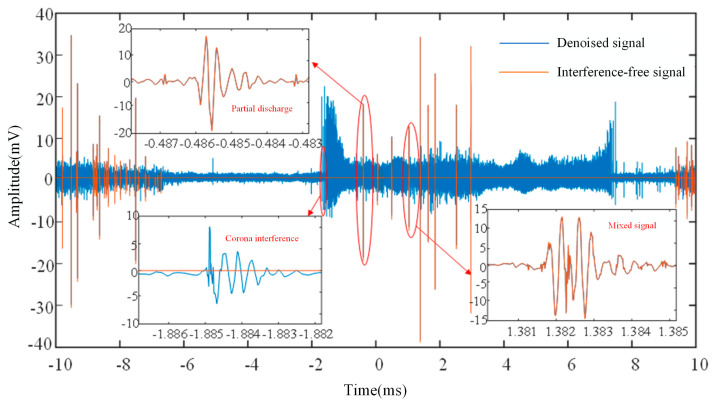
Mixed-signal separation at 27.5 kV based on WT-ST-2DCNN.

According to the mixed data application flow of the model above, the corona interference is separated from the long-time series signals measured at 27.5 kV mixed with corona interference and partial discharge, and the results are shown in Figure 17. The analysis results show that by correcting the position of the time window, the integrity of partial discharge and corona interference can be ensured in the process of sliding the interception signal on the long time sequence signal in the time window, which solves the problem that the corona interference is wrongly identified as partial discharge because the time window is truncated, and the pulse signal retained in the figure is a partial discharge signal. Figure 17 shows the experimental signals before and after the corona interference is removed, and the corona interference signal in the aliasing signal can be effectively eliminated by using the method in this paper to obtain the final partial discharge signal. Therefore, it can be concluded that WT-ST-2DCNN has a good separation effect on the partial discharge signal and corona interference in long-time sequence signals, and it has universal applicability.

## 4. Conclusions

In this paper, the wavelet algorithm is used to denoise the signal, and the partial discharge and corona interference are identified through the one-dimensional convolutional neural network. Then, a signal recognition algorithm combining ST and two-dimensional convolutional neural network is proposed to solve the problem in which the corona interference caused by the corona interference is truncated by the time window and is wrongly identified as partial discharge. Compared with the three time–frequency analysis methods, the signal recognition model of WT-ST-2DCNN was established, and the recognition effect of the model before and after wavelet noise reduction was tested. In the study of the separation of partial discharge and corona interference in long-time series signals, the time window is corrected, and the main conclusions are as follows:

- Compared with STFT, the ST overcomes the limitation of fixed time–frequency resolution, which means that it can provide variable resolution at different times and frequencies so as to better capture the local characteristics of the signal. At the same time, it also effectively avoids the problem of the wavelet basis and decomposition layer selection in wavelet transform, and it has more flexible time–frequency analysis ability.
- The noise reduction in the signal by WT removes the useless information on the time-frequency spectrum, which can better retain the local time-frequency characteristics of the signal, enhance the convergence speed and generalization ability of the model, and improve the classification accuracy of ST-2DCNN.
- The time position of the window is corrected based on the moment corresponding to the maximum energy in the ST matrix obtained from the ST. This ensures the integrity of PD and CD signals, effectively avoiding the misidentification of truncated corona interference as partial discharge. The results demonstrate that the WT-ST-2DCNN method exhibits excellent performance in separating partial discharge and pulse interference in long time-series signals.

Based on this paper, research work can be carried out from the following aspects in the future:

- Due to its depth and complexity, the 2DCNN model requires a lot of computing resources and time in the training process, and there are certain limitations for the actual field application. In the future, more efficient network structures, such as lightweight convolutional neural networks (such as MobileNet, ShuffleNet, etc.) can be studied to reduce the computational burden of the model and improve the real-time processing capabilities.
- Explore other feature extraction techniques, such as autoencoders or attention mechanisms, to further improve the feature learning ability of the model as well as the model’s recognition accuracy.
- A variety of detections are used for joint detection, combined with other sensor data, such as partial discharge ultrasonic signal, partial discharge UHF signal, etc., for multi-mode learning to improve the accuracy and robustness of partial discharge detection.

## Figures and Tables

**Table 1:** The 2DCNN parameter settings.

Parameters	Parameter Value
Number of iterations	500
Activation function	ReLU
Loss function	Cross entropy loss
Optimizer	Adam
Initial learning rate	0.01
Coefficients of L2 regularization terms	0.01
Learning rate decline factor	0.5
Learning rate decline cycle	450

## Data Availability

Data are contained within the article.
